# Characterisation of marine bacterium *Microbulbifer* sp. ALW1 with *Laminaria japonica* degradation capability

**DOI:** 10.1186/s13568-022-01482-y

**Published:** 2022-11-05

**Authors:** Zhipeng Li, Zeping Du, Hebin Li, Yanhong Chen, Mingjing Zheng, Zedong Jiang, Xiping Du, Hui Ni, Yanbing Zhu

**Affiliations:** 1 College of Ocean Food and Biological Engineering, Jimei University, Xiamen, 361021 China; 2 Fujian Provincial Key Laboratory of Food Microbiology and Enzyme Engineering, Xiamen, 361021 China; 3 Research Center of Food Biotechnology of Xiamen City, Xiamen, 361021 China; 4 Department of Pharmacy, Xiamen Medical College, Xiamen, 361008 China

**Keywords:** Brown algae, Polysaccharide degradation, *Microbulbifer*, Strain characteristics, Complete genome sequence

## Abstract

Marine bacterium *Microbulbifer* sp. ALW1 was revealed to be able to effectively degrade *Laminaria japonica* thallus fragments into fine particles. Polysaccharide substrate specificity analysis indicated that ALW1 could produce extracellular alginate lyase, laminarinase, fucoidanase and cellulase. Based on alignment of the 16 S rRNA sequence with other reference relatives, ALW1 showed the closest relationship with *Microbulbifer aggregans* CCB-MM1^T^. The cell morphology and some basic physiological and biochemical parameters of ALW1 cells were characterised. ALW1 is a Gram-negative, rod- or oval-shaped, non-spore-forming and non-motile bacterium. The DNA–DNA relatedness values of ALW1 with type strains of *M. gwangyangensis* (JCM 17,800), *M. aggregans* (JCM 31,875), *M. maritimus* (JCM 12,187), *M. okinawensis* (JCM 16,147) and *M. rhizosphaerae* (DSM 28,920) were 28.9%, 43.3%, 41.2%, 35.4% and 45.6%, respectively. The major cell wall sugars of ALW1 were determined to be ribose and galactose, which differed from other closely related species. These characteristics indicated that ALW1 could be assigned to a separate species of the genus *Microbulbifer*. The complete genome of ALW1 contained one circular chromosome with 4,682,287 bp and a GC content of 56.86%. The putative encoded proteins were categorised based on their functional annotations. Phenotypic, physiological, biochemical and genomic characterisation will provide insights into the many potential industrial applications of *Microbulbifer* sp. ALW1.

Key points.

## Key points

- *Microbulbifer* sp. ALW1 has* Laminaria japonica* degradation capability.
- Genomic information of strain ALW1 is useful for analysing the polysaccharides degradation process.
- *Microbulbifer* sp. ALW1 could be potentially applied in producing functional materials.

## Introduction

Marine macroalgae play a crucial part in the ecosystem. Seaweeds synthesise a wide variety of polysaccharides including alginate, agar, carrageenan, laminarin, fucoidan and cellulose, for use as cell wall components or storing nutrients (Jönsson et al. 2020). These polysaccharides can be exploited as feedstock for biofuel production and utilized as supplies for food, fertiliser, cosmetic and pharmaceutical products after further processing (Aswathi Mohan et al. 2022; Costa et al. 2021; Geetha Bai and Tuvikene 2021; Priyan Shanura Fernando et al. 2018). The worldwide consumption of seaweeds has been growing steadily owing to their health benefits, and seaweed aquaculture is experiencing a rapid expansion. Moreover, the amount of seaweed waste generated from the manufacturing process has increased, and the drifting seaweed debris from seaweed farming can cause marine eutrophication (Alemañ et al. 2019; Garcia-Poza et al. 2020; Jönsson et al. 2020). As such, the proper disposal and reutilisation of seaweed waste are important for environmental preservation and resource recovery (Lopez-Pedrouso et al. 2020). Utilising marine bacteria to accelerate the decomposition of seaweed waste has attracted great interests, as the complex structure of seaweed cell wall is not readily accessible to general microorganisms. The isolation and characterisation of specific bacterial strains that colonise on seaweed provide a feasible strategy to uncover novel bioresource for seaweed breakdown and to identify new enzymes contributing to degradation capability.

A collection of enzymes has been isolated from microorganisms and reported to function against the diverse polysaccharide constituents of seaweed cell wall, including alginate (Gao et al. 2021; Zhu et al. 2016b), agar (Park et al. 2020; Zhu et al. 2016b, c), carrageenan (Zhu et al. 2018), laminarin (Hu et al. 2021), fucoidan (Sichert et al. 2020) and cellulose (Li et al. 2021a). Complete genomic analysis has also been implemented to understand the genetic elements imparting the polysaccharide degradation ability of seaweed-associated bacteria (Sun et al. 2016; Zhu et al. 2016a).

The marine bacterial strain *Microbulbifer* sp. ALW1 has been isolated from rotten *Laminaria japonica* in our previous report (Zhu et al. 2016b). This study attempted to provide some phenotypic characteristics of strain ALW1 and to have an insight into its physiological and biochemical parameters. The genomic sequence analysis of strain ALW1 was chipped in to enrich our understanding of the genetic loci contributing to its capability of degrading diverse polysaccharides derived from seaweed cell wall. *Microbulbifer* sp. ALW1 could be potentially applied in reducing seaweed waste and producing functional materials from seaweeds. In addition, strain ALW1 could be utilised as a new genetic source of polysaccharide-degrading enzymes.

## Materials and methods

### Strain, materials and chemicals

*Microbulbifer* sp. ALW1 was deposited into the China Centre of Industrial Culture Collection (CICC) and the Japan Collection of Microorganisms (JCM) with accession numbers CICC 23,821 and JCM 33,586, respectively. Kelp (*L. japonica*) was purchased from a market in Xiamen, China. Sodium alginate was purchased from Sinopharm Chemical Reagent Co., Ltd. (Beijing, China). Laminarin and fucoidan were purchased from Shanghai Yuanye Biotechnology Co., Ltd. (Beijing, China). Carboxymethyl cellulose–sodium salt (CMC-Na) was purchased from Sigma-Aldrich (St. Louis, MO, USA).

### Bacterial cell culture and observations

The refreshed cells of strain ALW1 from our laboratory stock were sub-cultured in 400 mL of growth medium (30 g/L NaCl, 5 g/L [NH_4_]_2_SO_4_, 2 g/L K_2_HPO_4_, 1 g/L MgSO_4_·7H_2_O, 0.1 g/L FeSO_4_·7H_2_O, 5 g/L *L. japonica* thallus flakes) at 30 °C with stirring at 80 rpm for 72 h. The integrity of the thallus flakes was captured using a Canon 50D digital camera (Tokyo, Japan). The morphology of the thallus flakes was examined under a BA200 microscope of Motic China Group Co., Ltd. (Beijing, China).

### Preparation and polysaccharide degradation activity assay of crude extracellular enzymes

The ALW1 cells were cultured in the medium described above with 5 g/L fine particles of milled *L. japonica* thallus (380–830 μm in diameter) in place of the thallus flakes at 30 °C for 72 h with shaking at 180 rpm. The cell solution was centrifuged at 12,000×*g* for 15 min at 4 °C, and the supernatant was collected to determine the activities of extracellular enzymes from strain ALW1 against different polysaccharides. The polysaccharides tested included alginate, laminarin, fucoidan and cellulose. Enzyme activity was determined following the procedures of the 3,5-dinitrosalicylic acid (DNS) method (Hu et al. 2021; Jiang et al. 2019; Li et al. 2021a; Miller 1959). The reaction was initiated by adding 2 mL of the crude enzymes to 2 mL of 5 mg/mL individual substrate in 50 mM Na_2_HPO_4_-NaH_2_PO_4_ (pH 7.0). The substrates tested included sodium alginate, laminarin, fucoidan and CMC-Na. After incubation at 30 °C for 1 h, the reaction was stopped by adding 1 mL of DNS reagent. The reducing sugar was determined at 540 nm using the spectrophotometer of Varian Cary 50 (Palo Alto, USA). The unit of enzyme activity was defined as the amount of enzyme that released 1.0 µg of the reducing sugar (glucose equivalent) per hour under the measured conditions.

### Morphological, physiological and biochemical assays of the strains

After the cells were cultivated at 30 °C for 48 h on 2216E (Oppenheimer and ZoBell 1952) agar plate, the cells were subjected to cell morphology observations under a microscope of Olympus BH-2 light (Tokyo, Japan) and a scanning electron microscope of Hitachi SU8010 (Tokyo, Japan). Gram staining was performed following the standard procedures and was confirmed using the method of KOH lysis test (Gregersen 1978). The ALW1 cells grown in 2216E medium containing different NaCl concentrations (20–200 g/L) under different temperatures (4–55 °C) or pH (3.0–12.0) were monitored to examine the optimal growth conditions. Polar lipids were extracted by chloroform/methanol system and analysed according to the method of Huang et al. (2020). Extraction, separation and identification of fatty acid methyl esters were conducted following the instructions of the MIDI Microbial Identification System (MIDI, Inc., Newark, DE). Isoprenoid quinones were extracted from freeze-dried cells (200 mg) with chloroform/methanol (2:1) and analysed using PUMP 1525 reverse-phase high-performance liquid chromatography of Waters (Milford, USA). Oxidase and catalase activity assays were determined as described by Park et al. (2011). Hydrolysis of starch and Tween 80 was assayed by methods described by Bauer (1966). Physiological and biochemical characteristics were examined using API 50CH, API ZYM and API 20NE tests of bioMérieux (Marcy-L’etoile, France).

### 16 S rRNA analysis

The 16 S rRNA coding sequences of strain ALW1 (GenBank accession number KJ719305) and other validated species of genus *Microbulbifer* were aligned and analysed for phylogenetic relationship by neighbour-joining method with a bootstrap value of 1000 using the MEGA software (version 11) (Tamura et al. 2021).

### Genome sequencing and bioinformatic analysis

The genomic DNA of strain ALW1 was extracted using a bacterial genomic DNA extraction kit of Dongsheng Biotech Co., Ltd. (Guangzhou, China) according to the manufacturer’s instructions. The DNA–DNA hybridisation between strain ALW1 and the type strains from reference were carried out following the method described by Ley et al. (1970) using a Beckman DU 800 spectrophotometer (Miami, USA). A complete genome sequencing of *Microbulbifer* sp. ALW1 was conducted using the single-molecule real-time (SMRT) sequencing platform of PacBio Co., Ltd. (Menlo park, USA) (Faino et al. 2015). The genome was assembled with filtered SMRT subreads by employing the MHAP method. Protein coding sequences (CDSs) were predicted with Glimmer 3.02. (Delcher et al. 2007) MicroRNA (miRNA) genes were predicted by alignment with miRbase (Kozomara et al. 2018), rRNA genes were identified by scanning Rfam database (Kalvari et al. 2018), and tRNA genes were predicted by tRNAscan-SE (Chan and Lowe 2019). Annotations of the predicted genes were performed by alignment with the Clusters of Orthologous Genes (COG) (Galperin et al. 2017), Kyoto Encyclopaedia of Genes and Genomes (KEGG) (Kanehisa and Goto 2000), and Non-redundant (nr) databases using Basic Local Alignment Search Tool (BLAST) (Altschul et al. 1990). The families of structurally related catalytic and carbohydrate-binding modules of enzymes were searched including glycoside hydrolases (GHs), carbohydrate-binding modules (CBMs), glycosyltransferases (GTs), carbohydrate esterases (CEs), polysaccharide lyases (PLs), and auxiliary activities (AAs) on the basis of the CAZy database (Drula et al. 2022).

### Nucleotide sequence accession number

The complete genome sequence of *Microbulbifer* sp. ALW1 has been deposited in GenBank (National Center for Biotechnology Information), obtaining accession number CP047569.

## Results

### Digestion of algal cell wall polysaccharides by strain ALW1

**Fig. 1:**
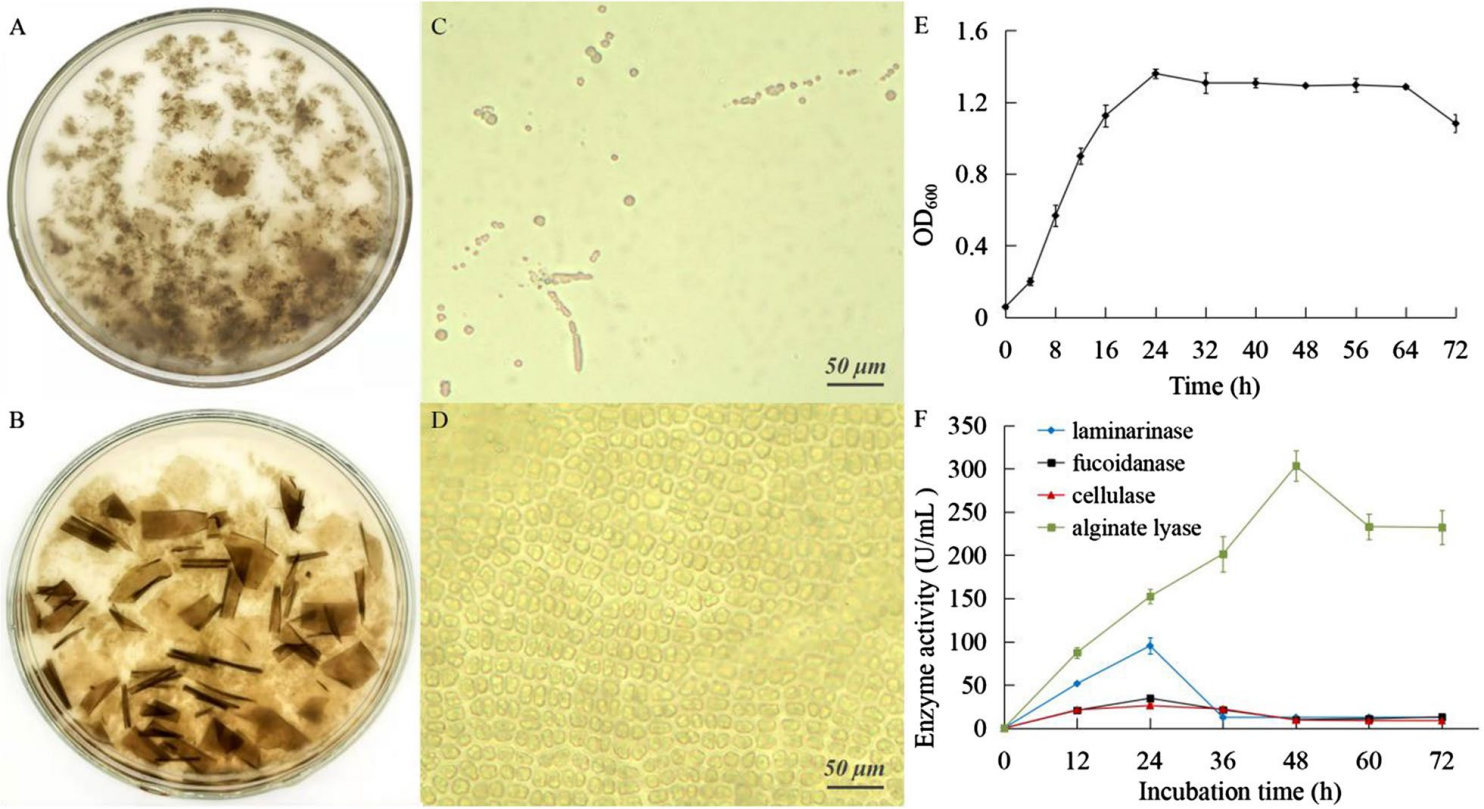
Degradation of kelp (*Laminaria japonica*) thallus fragments and polysaccharide substrates by strain ALW1 in kelp medium. **A** Kelp inoculated with the ALW1 cells. **B** Uninoculated kelp. **C** Micrograph of kelp cells inoculated with ALW1 strain and incubated at 30℃ at 80 rotation speed for 72 h, and kelp cells dispersed in the medium. **D** Micrograph of kelp cells in the culture medium without ALW1 strain and incubated at 30℃ with stirring at 80 rpm for 72 h, and the kelp cells were still neatly arranged. **E** Growth of strain ALW1 in *L. japonica* medium at 30 °C with stirring at 80 rpm for 72 h. **F** Extracellular polysaccharide-degrading enzymes’ activities. Enzyme activity analysis included alginate lyase, laminarinase, fucoidanase and cellulase assays. All the above enzyme activities were measured after ALW1 strain was inoculated in kelp medium and incubated at 30℃ with stirring at 80 rpm for 72 h. Data are presented as mean ± SD from three independent experiments

After *L. japonica* thallus fragments and the ALW1 cells were co-incubated for 72 h, the thallus fragments were broken down into the finest particles (Fig. 1A). In comparison, the thallus fragments that were not inoculated still remained intact (Fig. 1B). The close-up view of the undigested thallus fragments showed abundant multi-cellular sheets (Fig. 1D). By contrast, numerous fine circular algal particles in the medium were produced by the thallus fragments incubated with the ALW1 cells (Fig. 1C). The effective disintegration of brown algal cell wall by strain ALW1 suggested that the ALW1 cells could secrete degradation enzymes to decompose structural polysaccharides. The growth of ALW1 cells could achieve mid-log phase at 12 h after inoculation and arrive at stationary phase at 24 h after inoculation (Fig. 1E). The polysaccharide degradation activities of ALW1 cells were supported by the observations that the crude extracellular enzymes could digest the polysaccharide substrates of sodium alginate, laminarin, fucoidan and CMC-Na. In the time–course activity assays, the secretion of ALW1 cells demonstrated the highest enzyme activity against sodium alginate, followed by laminarin, fucoidan and CMC-Na, with peaks at 303.4, 95.3, 34.5 and 26.0 U/mL, respectively (Fig. 1 F). The most active alginate lyase activity of ALW1 cells was achieved in the cell culture at 48 h, whereas laminarinase, fucoidanase and cellulase activities were most active at 24 h of cell culture. The activity of crude enzymes against CMC-Na was substantially lower than those against the other substrates.

### Strain ALW1 identification by phylogenetic relationship and DNA-DNA hybridisation analysis

**Fig. 2:**
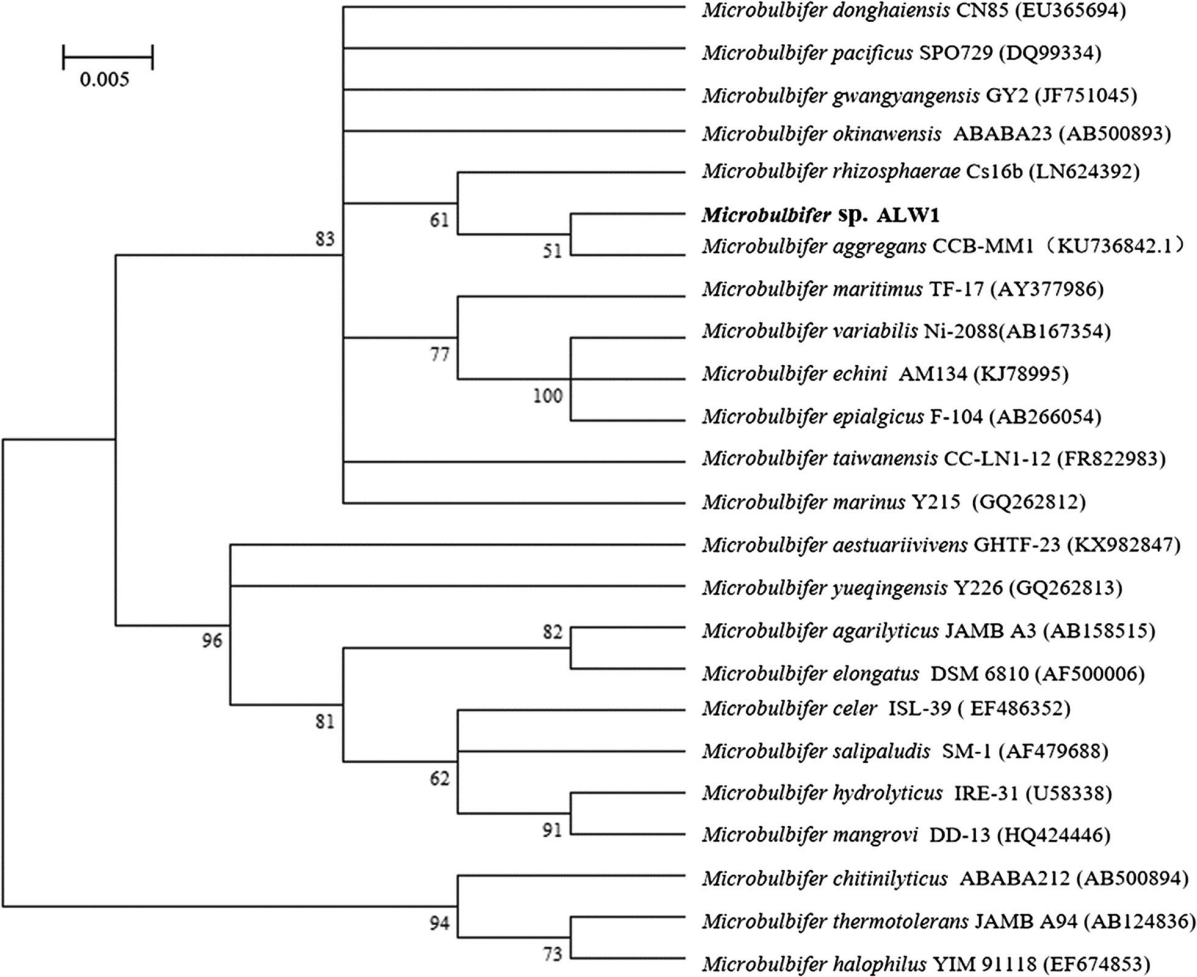
Phylogenetic tree analysis for strain ALW1 and its closely related species within genus *Microbulbifer*. Numbers at the nodes indicate the percentages of bootstrap support based on neighbour-joining analysis of 1000 re-sampled datasets. Bar, 0.005 substitutions per nucleotide position

The phylogenetic relationship of 16 S rRNA sequence indicated that strain ALW1 was more closely related to *M. aggregans* CCB-MM1^T^ and *M. rhizosphaerae* Cs16b^T^ than other related species of genus *Microbulbifer* (Fig. 2). The top hits on the sequence similarity to strain ALW1 were *M. rhizosphaerae* Cs16b^T^ (98.3%), *M. aggregans* CCB-MM1^T^ (97.7%), *M. okinawensis* ABABA23^T^ (97.7%), *M. maritimus* TF-17^T^ (97.5%) and *M. gwangyangensis* GY2^T^ (97.3%). The DNA–DNA hybridisation relatedness values between strain ALW1 and the type strains were 45.6% (*M. rhizosphaerae* DSM 28,920), 43.3% (*M. aggregans* JCM 31,875), 35.4% (*M. okinawensis* JCM 16,147), 41.2% (*M. maritimus* JCM 12,187) and 28.9% (*M. gwangyangensis* JCM 17,800), implying that strain ALW1 is a member of a genomic species different from the reference species of genus *Microbulbifer*.

### Morphological, physiological and biochemical characteristics of ALW1 cells

**Fig. 3:**
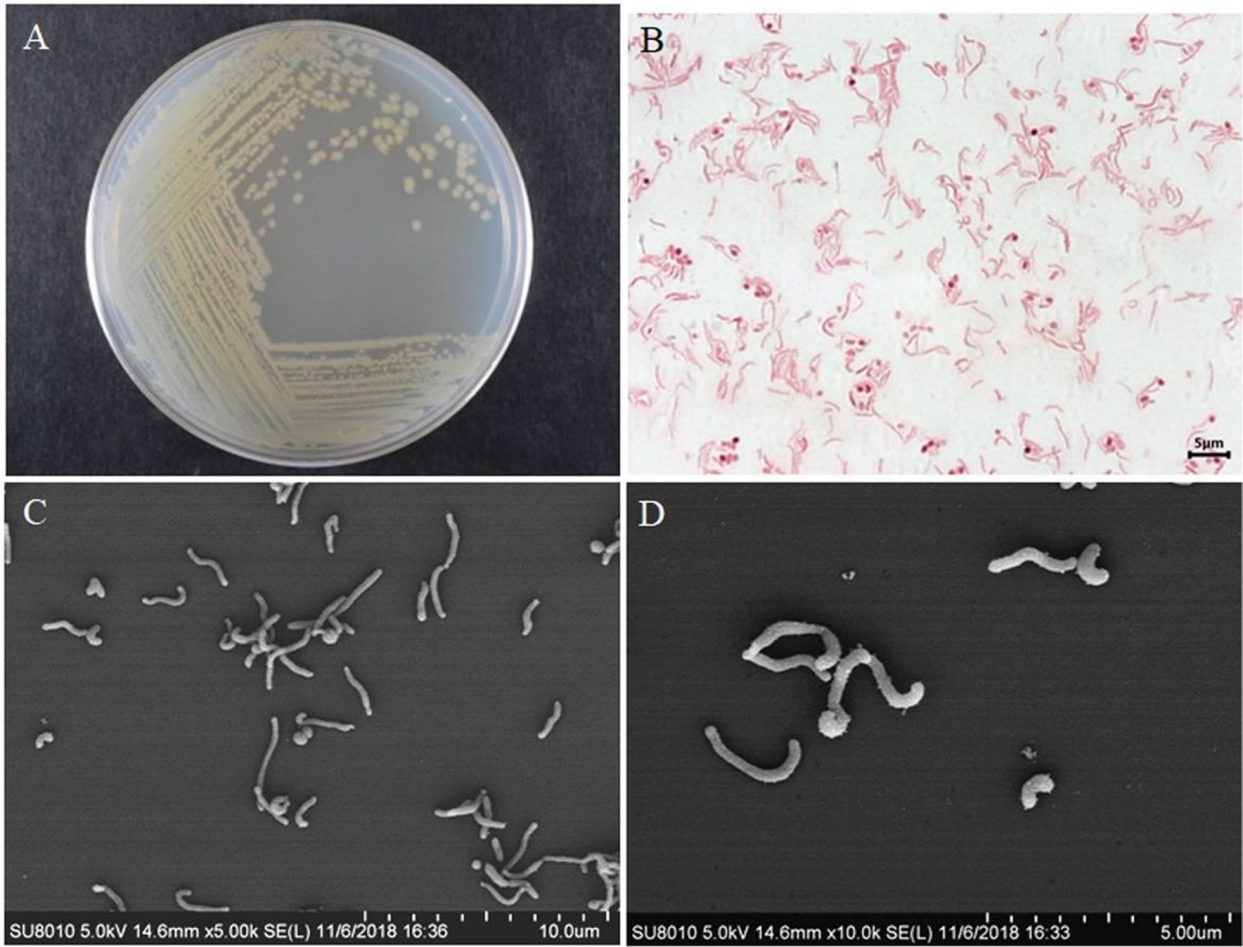
Morphological observations of strain ALW1. **A** Colonies of ALW1 cells on 2216E agar plate. **B** Light microscope observation of strain ALW1. **C** and **D** Scanning electron microscope observation of strain ALW1. Bars, 10 μm (**C**) and 5 μm (**D**)

After colonies of ALW1 cells were incubated at 30 °C for 48 h on 2216E agar plate, the cells were circular to slightly irregular, opaque and yellow, and had a wet surface (Fig. 3A). Gram stain indicated that ALW1 was Gram negative. Microscopic examination indicated that straining ALW1 was rod shaped (0.2–0.4 μm × 0.9–3.9 μm) or oval shaped (0.4–0.5 μm × 0.4–0.6 μm, Fig. 3B–D), non-motile and non-spore forming. The ALW1 cells could grow at 10–45 °C, pH 5.0–10.0 and in 2216E medium containing 2.0–10.0% (w/v) NaCl. Optimal growth was found at 30 °C, pH 6.0–7.0 and with 2.0% (w/v) NaCl. The API 50CH test showed that the ALW1 cells were positive for esculin, 5-keto-gluconate, D-lactose and D-toulon sugars. The API ZYM profile indicated that the ALW1 cells were positive for alkaline phosphatase, esterase (C4), lipid esterase (C8), leucine arylamidase, valine arylamidase, acid phosphatase and naphthol-AS-BI-phosphohydrolase; weakly positive for lipase (C14) and cystine arylamidase; and negative for trypsin, chymotrypsin, α-galactosidase, β-galactosidase, β-glucuronidase, α-glucosidase, β-glucosidase, *N*-acetylglucosaminase, β-fucosidase and α-mannosidase. In the API 20NE test, ALW1 could utilise glucose, arabinose, *N*-acetylglucosamine and D-maltose. It could hydrolyse aescin and gelatin. The main quinone was ubiquinone-8 (100%). The main polar lipids were phosphatidyl glycerol, phosphatidyl ethanolamine, unidentified aminolipids (UAL 1–2), unidentified phospholipid, unidentified lipid, glycolipids (GL 1–2) and unidentified phosphatidyl glycolipid. The major fatty acids of strain ALW1 were C_18:1_*ω*7*c*/C_18:1_*ω*6*c*, iso-C_15:0_, C_16:0_ 10-methyl/iso-C_17:1_*ω*9*c* and C_16:1_*ω*7*c*/C_16:1_*ω*6*c*. The comparison of the physiological and biochemical characteristics between ALW1 and the type strains of closely related species of genus *Microbulbifer* were summarised in Table 1. Although the cell wall characteristic component of strain ALW1 and other related strains were meso-diaminopimelic acid, the main sugar components in cell wall of strain ALW1 were ribose and galactose, which were different from other closely related species. All of the above data confirmed that strain ALW1 could be assigned to a separate species of genus *Microbulbifer*.

**Table 1:** Characteristics of strain ALW1 and some type strains of closely related species of the genus *Microbulbifer*

Characteristic	ALW1	JCM 17,800	JCM 31,875	JCM 12,187	JCM 16,147	DSM 28,920
Temperature for growth (°C)						
Range	10–45	10–40	15–45	15–45	10–45	15–45
Optimum	30	30–35	30–35	30–35	35	30–35
pH						
Range	5.0–10.0	5.0–9.0	5.0–9.0	5.0–10.0	5.0–10.0	6.0–10.0
Optimum	6.0–7.0	7.0	7.0–8.0	7.0	6.0–7.0	7.0–8.0
NaCl (%)						
Range	2.0–10.0	2.0–8.0	2.0–14.0	2.0–10.0	2.0–10.0	2.0–8.0
Optimum	2.0	2.0	2.0	2.0–4.0	2.0	2.0
API 50CH						
D-Galactose	–	–	–	–	–	–
D-Glucose	–	–	–	–	–	–
D-Mannose	–	–	–	–	–	–
L-Rhamnose	–	–	–	–	–	–
D-Cellobiose	–	–	–	–	+	–
D-Maltose	–	–	–	–	–	–
D-Lactose	+	–	–	–	–	–
D-Melibiose	–	–	–	–	–	–
D-Sucrose	–	–	–	–	–	–
D-Trehalose	–	–	–	–	–	–
API ZYM						
Alkaline phosphatase	+	+	+	+	+	+
Lipase (C14)	w	w	w	w	+	w
Valine arylamidase	+	+	+	+	+	+
Cystine arylamidase	+	+	–	–	+	–
Chymotrypsin	–	–	–	–	+	–
α-Glucosidase	–	–	–	–	–	–
N-Acetylglucosaminase	–	–	–	–	–	–
API 20NE						
Nitrate reduction	–	+	–	–	–	–
Aescin hydrolysis	+	+	+	–	+	+
Gelatin hydrolysis	+	+	–	+	+	+
Glucose utilization	+	+	+	+	–	–
Mannose utilization	–	–	–	–	–	–
D-Maltose utilization	+	+	+	+	+	–
Malic acid utilization	–	–	+	+	+	–
Others						
Oxidase	w	+	w	–	–	+
Catalase	w	+	+	w	w	–
Starch hydrolysis	+	+	+	+	+	–
Quinone composition	Q-8 (100%)	Q-8 (100%)	Q-8 (100%)	Q-8 (100%)	Q-8 (100%)	Q-8 (100%)
Major fatty acids	Summed feature 8 (C_18:1_*ω*7*c*/C_18:1_*ω*6*c*, 20.29%), iso-C_15:0_ (14.67%), Summed feature 9 (C_16:0_ 10-methyl/iso-C_17:1_*ω*9*c*, 13.87%), Summed feature 3 (C_16:1_*ω*7*c*/C_16:1_*ω*6*c*, 12.07%)	iso-C_11:0_ 3-OH (19.13%), iso-C_15:0_ (15.94%), Summed feature 9 (C_16:0_ 10-methyl/iso-C_17:1_*ω*9*c*, 14.99%), iso-C_11:0_ (14.94%)	Summed feature 9 (C_16:0_ 10-methyl/iso-C_17:1_*ω*9*c*, 25.92%), iso-C_15:0_ (18.51%)	iso-C_11:0_ 3-OH (18.34%), iso-C_15:0_ (13.80%), Summed feature 9 (C_16:1_ 10-methyl/iso-C_17:1_*ω*9*c*, 13.46%)	iso-C_15:0_ (18.76%), Summed feature 9 (C_16:0_ 10-methyl/iso-C_17:1_*ω*9*c*, 17.70%), iso-C_11:0_ 3-OH (12.74%), iso-C_11:0_ (10.08%)	iso-C_11:0_ 3-OH (26.70%), iso-C_15:0_ (14.67%), iso-C_11:0_ (13.69%)
Cell wall sugar components	Ribose, galactose	Ribose, glucose	Ribose	Ribose, glucose	Ribose, galactose, glucose, mannose	Ribose, glucose
Polar lipid profile^†^	PG, PE, UAL 1–2, UPL, UL, GL 1–2, UPGL	PG, PE, UAL 1–4, UL 1–3, GL 1–2, UPGL 1–2	PG, PE, UAL 1–2, UPL, UAPL, GL 1–2, UPGL 1–2	PG, PE, UAL 1–4, UAPL, UL 1–4, GL 1–2, UPGL	PG, PE, UAL 1–2, GL 1–2, UPGL 1–2	PG, PE, UAL, UAPL, GL 1–2, UPGL, UL 1–2

Strains: ALW1, *Microbulbifer* sp. ALW1; JCM 17,800, *Microbulbifer gwangyangensis* JCM 17,800; JCM 31,875, *Microbulbifer aggregans* JCM 31,875; JCM 12,187, *Microbulbifer maritimus* JCM 12,187; JCM 16,147, *Microbulbifer okinawensis* JCM 16,147; DSM 28,920, *Microbulbifer rhizosphaerae* DSM 28,920. +, positive; –, negative; w, weakly positive. †* PE* phosphatidyl ethanolamine,* PG* phosphatidyl glycerol,* GL* glycolipids,* UL* unidentified lipid,* UAL* unidentified aminolipids,* UPL* unidentified phospholipid,* UPGL* unidentified phosphatidyl glycolipid,* UAPL* unknown aminophospholipid

### Genomic dissection of strain ALW1

**Fig. 4:**
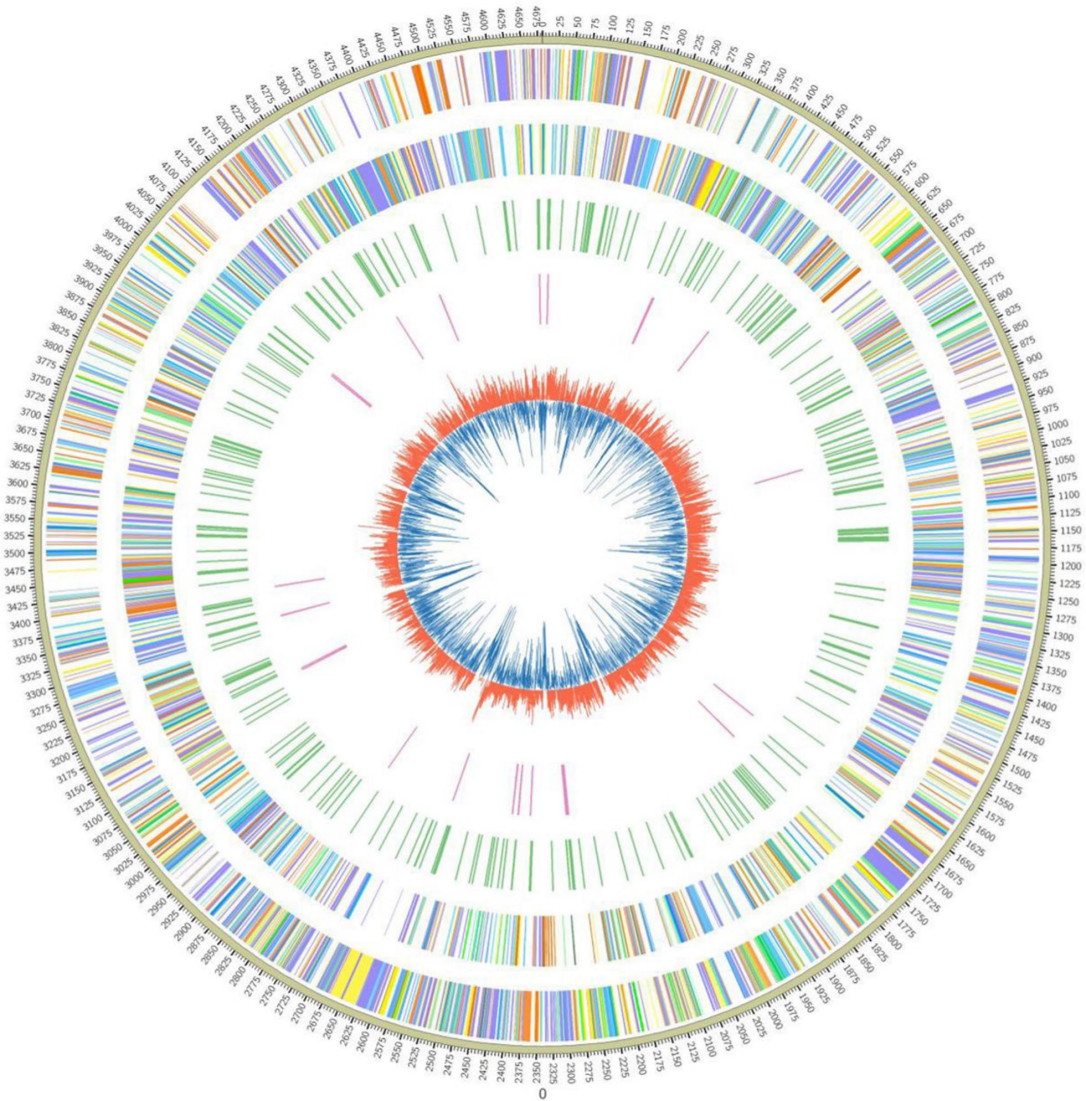
Genome sequence of *Microbulbifer* sp. ALW1. The outermost circle is the mark of genome size, and each scale is 0.1 MB. The second and third circles are genes on the positive and negative chains of the genome respectively. Different colors represent different cog functional classifications. The fourth circle is the repeat sequence. The fifth circle is tRNA. The innermost layer is the GC content. The red part indicates that the GC content in this region is higher than the average GC content of the genome. The higher the peak, the greater the difference from the average GC content. The blue part indicates that the GC content in this region is lower than the average GC content of the genome. 0 represents the conting number

The complete genome of *Microbulbifer* sp. ALW1 contained one circular chromosome with 4,682,287 bp and a GC content of 56.86% (Fig. 4). 3767 protein-coding genes were predicted, averaging 1,092 bp in length. Out of the putative proteins, 3599 had matched sequences in the nr database. Functional annotations suggested that 2671 and 1905 proteins had functional assignments based on their similarities to COG and KEGG groups, respectively. In addition, 38 rRNA, 30 tRNA and 3 miRNA genes were identified in the genome of strain ALW1.

### Putative CAZymes of strain ALW1

**Fig. 5:**
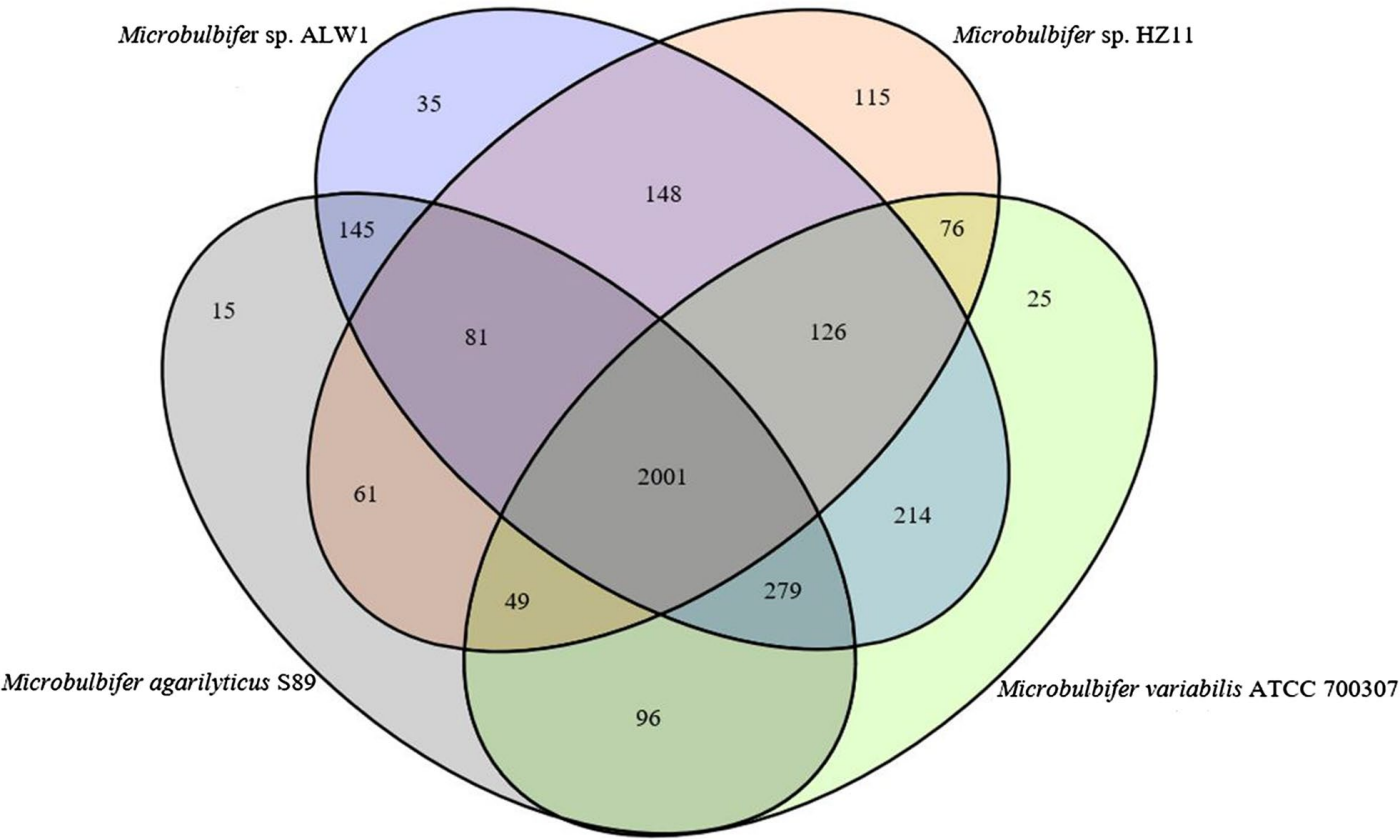
CAZyme classification of the putative proteins of *Microbulbifer* sp. ALW1. AA means auxiliary activity. CBM means carbohydrate binding module. CE means carbohydrate esterase. GH means glycoside hydrolases. GT means glycosyltransferases. PL means polysaccharide lyase

CAZyme analysis was conducted for the putative proteins of strain ALW1 to further understand the seaweed-degrading ability of this strain. For the putative carbohydrate active enzymes of strain ALW1, the numbers of GH, CBM, CE, GT, AA and PL were 81, 59, 40, 33, 17 and 14, respectively (Fig. 5). The GH number was predominant over the others, and the PL number was the least abundant.

## Discussion

In this study, *Microbulbifer* sp. ALW1 could produce extracellular alginate lyase, laminarinase, fucoidanase and cellulase. The dynamics of these polysaccharide degradation enzymes were consistent with the ingredients in brown algal cell wall. Similarly, *Microbulbifer* sp. 6532 A is able to degrade fragments of Wakame thallus, alginate and cellulose (Wakabayashi et al. 2012). The cell wall of *L. japonica* includes alginate, laminarin and fucoidan (Li et al. 2022a). The reducing sugars produced during incubation with ALW1 cells were probably utilised by the strain to support the cell growth. The ALW1 cells or its related enzymes could be used to produce oligosaccharides or monosaccharides (Hu et al. 2021; Jiang et al. 2019; Li et al. 2022b; Zhu et al. 2016b). Algal oligosaccharides have antitumour, antihypertensive, antioxidant and whitening effects, and they also can suppress IgE production, improve the intestinal microflora, and promote cell proliferation and plant growth (Chen et al. 2019; Jagtap et al. 2022; Zhu et al. 2021). Strain ALW1 might be suitable for the degradation and reduction of algal wastes to produce functional oligo- or monosaccharides.

**Fig. 6:**
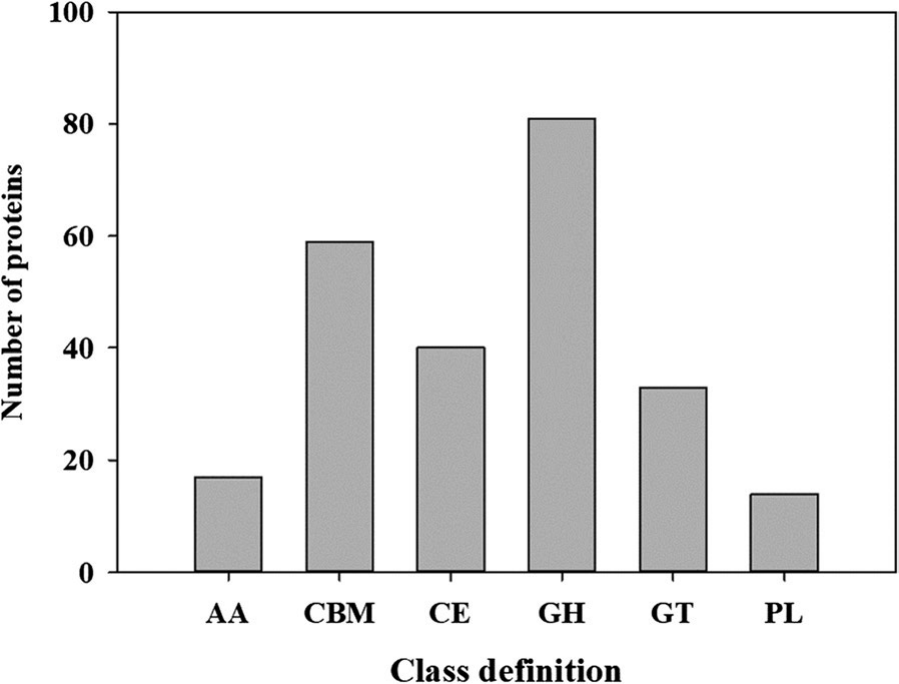
Venn diagram illustrating the genes shared by the *Microbulbifer* sp. strains ALW1, S89, HZ11, and ATCC 700,307

The phylogenetic relationship, DNA–DNA hybridisation and strain characteristics analyses indicated that strain ALW1 could be assigned to a separate species of genus *Microbulbifer*. Genus *Microbulbifer* is extensively applied in related industries owing to its ability to degrade seaweed or seaweed polysaccharides producing bioethanol or active oligosaccharides (Imran et al. 2017; Sun et al. 2014; Yang et al. 2018; Zhu et al. 2016b). The size of the *Microbulbifer* sp. ALW1 chromosome was roughly equal to those of *Microbulbifer* sp. HZ11 (GenBank: JELR00000000.1), *Microbulbifer agarilyticus* S89 (GenBank: AFPJ00000000.1) and *Microbulbifer variabilis* ATCC 700,307 (GenBank: AQYJ00000000.1), which can degrade algal polysaccharides (Lee et al. 2017; Oh et al. 2011; Sun et al. 2014). Automated genome comparison suggested that the chromosome of *Microbulbifer* sp. ALW1 was widely collinear with those of *Microbulbifer* sp. HZ11 and ATCC700307 (Fig. 6). The four strains shared a common core genome of 2001 genes (Fig. 6). The CDSs of *Microbulbifer* sp. ALW1 and ATCC700307 were highly similar, and more species-unique genes were detected in the CDSs of *Microbulbifer* sp. HZ11 (Fig. 6). The genome sequence of *Microbulbifer* sp. ALW1 will facilitate a better understanding of the molecular mechanism of brown algae metabolism by this strain and provide insight into the potential biotechnological applications of strain ALW1 in various fields, such as food, cosmetic, pharmaceutical, biofuel and fertiliser manufacturing industries.

GHs are involved in the hydrolysis and/or transglycosylation of glycosidic bonds present in glycosides, glycans and glycoconjugates (Berlemont and Martiny 2016), participating in the metabolism of cellulose, laminarin, fucoidan, and other polysaccharides (Berlemont and Martiny 2016; Hu et al. 2021; Jagtap et al. 2022; Nguyen et al. 2018). Some GH enzymes from *Microbulbifer* sp. ALW1 have been characterised in our previous work, including a novel laminarinase MaLamNA (Hu et al. 2021), a β-glucosidase MaGlu1A belonging to GH1 (Jiang et al. 2021), and an endo-β-1,4-glucanase MaCel5A belonging to GH5 (Li et al. 2021a). Polysaccharide lyases have substrate specificity and can break the O–C4 bond to the uronic acid using a β-elimination mechanism (Li et al. 2021b). Five putative alginate lyase coding genes were found in the genome of *Microbulbifer* sp. ALW1. These alginate lyases could be used for many applications, such as the production of active oxygen species, preparation of algal protoplasts, analysis of alginate structure, and medical treatment of cystic fibrosis. A new gene of exo-oligoalginate lyase AlgL17 belonging to PL17 has been cloned from strain ALW1 and characterised in our previous study (Jiang et al. 2019). The substrate preference of the catalytic domain divides PLs into several families (Li et al. 2021b). CBMs play key roles in the recognition and binding processes of polysaccharide substrates and enzymes during the degradation of algal polysaccharides (Guillen et al. 2010; Sidar et al. 2020).

**Fig. 7:**
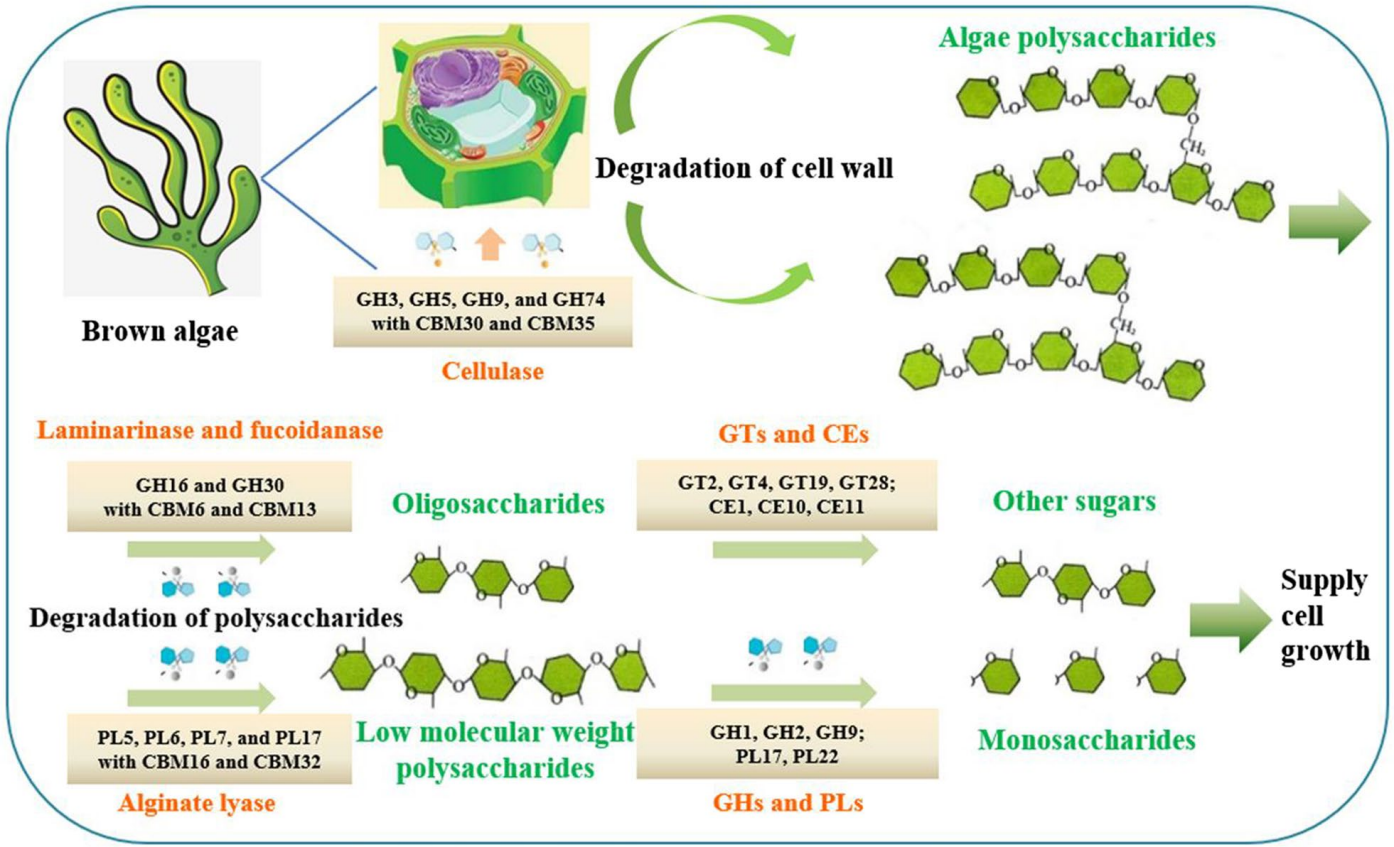
Proposed degradation process of brown algae polysaccharides by *Microbulbifer* sp. ALW1

Based on the abundance of CAZymes, the process of seaweed degradation through the action of cohort enzymes in strain ALW1 was proposed (Fig. 7). Through the action of GHs and CBMs in *Microbulbifer* sp. ALW1, common carbohydrates in nature, such as cellulose of the seaweed cell wall components, would be destroyed through cleavages (Li et al. 2021a; Zhang et al. 2021), and a large amount of seaweed polysaccharides would be released. CBM30 and CBM35 would be important modules in this process. Some GHs (Hu et al. 2021; Kusaykin et al. 2016; Sichert et al. 2020) and PLs (Li et al. 2021b; Zhu et al. 2016b), such as laminarinase, fucoidanase and alginate lyase, would destroy seaweed polysaccharides through cleavages and release a large amount of low-molecular-weight polysaccharides, oligosaccharides and monosaccharides. CBM6, CBM13, CBM16 and CBM32 acting as the associated constructions would play important roles in the recognition and binding processes of substrates and enzymes. The low-molecular-weight polysaccharides and oligosaccharides could further be degraded into monosaccharides by GHs and PLs or transformed into other sugars by GTs and CEs (Chen et al. 2018). These monosaccharides could provide supplies for cell energy metabolism and basic growth needs.

To summarise, the marine bacterial strain *Microbulbifer* sp. ALW1 characterised in this study was capable of degrading brown algal cell wall polysaccharides, including alginate, laminarin, fucoidan and cellulose. The physiological and biochemical characteristics of strain ALW1 suggested that it might be a novel species of genus *Microbulbifer*, distinct from previously annotated reference species. The genetic information of strain ALW1 and the predicted carbohydrate active enzymes responsible for the polysaccharides’ metabolism enriched our understanding of the bioactivity of strain ALW1 and provided a strong support for its industrial utilisation for algal waste cleanup and bioresource recovery.

## Data Availability

All data generated and analyzed during this study are included in this published article.
